# Micro-fragmented adipose tissue injection associated with arthroscopic procedures in patients with symptomatic knee osteoarthritis

**DOI:** 10.1186/s12891-018-2105-8

**Published:** 2018-05-30

**Authors:** G. Cattaneo, A. De Caro, F. Napoli, D. Chiapale, P. Trada, A. Camera

**Affiliations:** 1 Spotorno Foundation c/o San Michele Clinics, Viale Pontelungo, 79, 17031 Albenga, SV Italy; 2 Orthopaedic Department, San Paolo Hospital, Savona, SV Italy

**Keywords:** Knee chondropathy, Osteoarthritis, MSCs, ASCs, Micro-fragmented adipose tissue

## Abstract

**Background:**

The social impact of degenerative diseases is steadily increasing, because of the continued rise in the mean age of the active population. Articular cartilage lesions are generally associated with disability and symptoms such as joint pain and reduced function, and remain a challenge for the orthopaedic surgeon. Several non-invasive solution have been proposed, but the results achieved to date are far from being completely satisfactory. Recently, new therapeutic approaches, such as the use of mesenchymal stem cells, have been developed. Among the many sources, the adipose tissue is nowadays considered one of the smartest, due to its abundance and easy access. The aim of this retrospective study is to explore whether patients affected by symptomatic knee osteoarthritis treated with micro-fragmented adipose tissue associated with a chondral shaving procedure experience an improvement in symptoms and function.

**Methods:**

Thirty-eight patients affected by symptomatic knee osteoarthritis were treated in 2015 with an arthroscopic procedure associated with an injection of autologous and micro-fragmented adipose tissue. Micro-fragmented adipose tissue was obtained using a minimal manipulation technique in a closed system. Clinical outcomes were determined at 1, 3, 6, and 12 months follow-up using Knee Injury and Osteoarthritis Outcome Score questionnaire and direct physical examination. Safety of the procedure, recording type and incidence of any adverse event, was also assessed.

**Results:**

A steady and statistically significant improvement of all the clinical scores from pre-operative evaluation to 1, 3, 6, and 12 months follow-up was observed, with KOOS sport and quality of life being the most improved scores. On average, 92% of the patients clinically improved and 100% of them were satisfied with the treatment. No adverse events nor relevant complications were recorded.

**Conclusion:**

The result of the study pointed to micro-fragmented adipose tissue as a safe and beneficial adjuvant in the surgical treatment of degenerative knee chondropathy. The procedure is simple, sustainable, quick, minimally invasive, one-step, and safe. After one year, the results are very satisfactory and promising. A longer follow-up is needed to draw definitive conclusions and enlarge the indications.

**Trial registration:**

Registered at clinicaltrials.gov as NCT03527693 on 27 April 2018 (retrospectively registered).

## Background

The social impact of degenerative diseases such as articular cartilage pathology is steadily increasing, because of the continued rise in the mean age of the active population. Articular cartilage lesions are generally, but not always, associated with disability and with symptoms such as joint pain and reduced function, and might progress to end-stage osteoarthritis (OA). Varieties of non-invasive solutions for pain relief, improvement in function and disability, and eventually, modification of the progression of severe cartilage lesions and OA, have been proposed with variable success rates [1–3]. Non-surgical treatments, such as physiokinesitherapy, pharmacological treatments (analgesics/anti-inflammatory agents, glucocorticoids, opioids, nonsteroidal anti-inflammatory drugs - NSAIDs), hyaluronic acid (HA) or its derivatives [4, 5] or platelet-rich plasma (PRP) injections [6, 7], indicated for small or diffused degenerative lesions, temporarily target the symptoms but cannot prevent the degeneration process [8]. Joint-preserving surgical treatments, such as arthroscopic shaving or debridement, laser chondroplasty, and microfractures, provide temporary relief of symptoms, but the clinical findings are highly variable and many authors contraindicated their use for diffused joint degeneration. For these reasons, new therapeutic approaches, such as the use of mesenchymal stem cells (MSCs) have been developed. Through trophic, mitogenic, anti-scarring, anti-apoptotic, immunomodulatory, and anti-microbial actions, produced by a large amount of bioactive elements, growth factors and cytokines, MSCs “sense” and “signal” changes in the microenvironment where they reside [9, 10]. Bone marrow and adipose tissue are the most readily available sources of MSCs, and, in this context, the adipose tissue is nowadays considered the smartest due to its abundance, the easy access and the simple isolation procedure [11, 12]. In addition, of the many cell types contained in the adipose tissue, MSCs (ASCs) comprise up to 2%, whereas only 0.02% of cells in bone marrow are MSCs. The use of ASCs, either culture-expanded or obtained by mechanical or enzymatic treatment as stromal vascular fraction (SVF) [13] have recently created a huge interest in the context of cartilage regeneration and shown promising results [14–18]. However, the studies published to date used a tissue-engineering approach, involving the use of scaffolds, cells, and growth factors, either alone or in any combination [16, 19–21]. In addition to the large number of processing steps, the high economic burden, and the restrictions associated with cell expansion and extensive manipulation [22, 23], the results achieved to date are far from being completely satisfactory. Therefore, availability of a minimally manipulated adipose tissue providing in one-step the key elements to support a natural regenerative response would have remarkable clinical relevance [24]. Based on these considerations, a commercially available technique that intra-operatively provides micro-fragmented and minimally manipulated adipose tissue without expansion or enzymatic treatment [25] was employed in this study. This approach, that has been already shown to be safe and promising in different pathologies [26–33], provides the key elements to support a natural reparative response, that is scaffold (the adipose tissue structure), cells (ASCs), and growth factors (secreted cytokines and chemokines) [34]. The aim of this retrospective study is to explore whether patients affected by symptomatic knee OA treated with micro-fragmented adipose tissue injection associated with a chondral shaving procedure experience an improvement in symptoms and function.

## Methods

### Study population

The retrospective analysis of the patient data was approved by the Regional Ethics Committee of Liguria - Italy (protocol n° 164REG2016, September 22, 2016). No approval for the initial treatment of the patients was required being the procedure standard clinical practice in our Hospital. In 2015, 38 consecutive patients affected by symptomatic knee OA underwent an arthroscopic procedure associated with an injection of autologous and micro-fragmented adipose tissue. The indication for the treatment was knee chondropathy grade > II (ICRS classification), constant pain and failure of conservative treatments (physiokinesitherapy, corticosteroids, HA and/or PRP) for at least 12 months. Contraindications for the treatment were immune-mediated (non-infectious) synovitis, OA Kellgren-Lawrence grade > 3, axial defects > 10°, metabolic disorders and BMI > 40. Pre-operative assessments included standard X-rays, MRI, direct physical examination, Knee Injury and Osteoarthritis Outcome Score (KOOS) questionnaire. Of these 38 patients, 3 subjects were treated with micro-perforations because of a severe chondral damage and where excluded from the global analysis. The remaining 35 underwent a standard chondral shaving procedure [35]. Those patients having also a meniscal injury no longer repairable (14 patients) underwent an associated meniscectomy to correct the defect.

### Harvesting of the adipose tissue

The lower or the lateral abdomen was chosen as donor site for adipose tissue harvesting. Before harvesting the fat, the site was injected with Klein solution (1 vial adrenaline + 50 cm^3^ mepivacaine 2% in 250 cm^3^ saline) using a disposable 17G blunt cannula connected to a luer-lock 60-cm^3^ syringe. The fat was then harvested using a 13G blunt cannula, for a fast and a-traumatic suction, connected to a VacLok® 20-ml syringe.

### Processing of the adipose tissue with Lipogems® device and injection in the joint

The harvested fat was immediately processed in the Lipogems® processing kit, a disposable device that progressively reduces the size of the adipose tissue clusters while eliminating oily substances and blood residues with pro-inflammatory properties. The entire process, carried out in one surgical step, was performed in complete immersion in physiological solution minimizing any trauma to the cells. The resulting micro-fragmented fat was collected in a 60-cm^3^ syringe and positioned for decanting the excess saline solution. The resulting product was then transferred into several 10-cm^3^ syringes to be injected in the patient. Micro-fragmented fat (10 cm^3^) was injected intra-articular after the arthroscopic procedure (shaving or shaving + meniscectomy).

### Post-op rehabilitation protocol and clinical evaluations

All patients were discharged the day after the procedure with an elastic compression band on the harvesting site, low MW heparin for 3 weeks, 2 weeks of unloading and then full load recovery in the following 7 days. Continuous passive motion from the immediate post-op and active physiokinesitherapy from day 15 post-op.

Standard clinical evaluations at 1, 3, 6, and 12 months post-op included Knee Injury and Osteoarthritis Outcome Score (KOOS) questionnaire and direct physical examination with the evaluation of knee range of motion, patellar subluxation, ability to walk, go up and down the stairs, squatting, muscular strength, stiffness and knee swelling. The Western Ontario and McMaster Universities Osteoarthritis Index (WOMAC) was calculated from the KOOS.

### Safety assessment

Safety was assessed by evaluating local adverse events, such as infections, fever, and excessive swelling of the knee at 1, 3, 6, and 12 months post-op.

### Statistical analysis

To guarantee standard operating procedures, all the patients were operated by the same surgeon (GC) and the clinical evaluations at any follow-up time were performed by the same surgeon assisted by another surgeon. Considering the relatively small sample size, results are expressed as the mean and standard deviation. For statistical comparisons, the chi-squared test for all categorical data, Student’s t-test for unpaired groups for parametric data, and Mann-Whitney test for non-parametric data (calculated with the Kolmogorov-Smirnoff normality test) were used (GraphPad Prism v5.0, La Jolla, USA). A *p* < 0.05 was considered statistically significant. A *p* < 0.1 is reported as a tendency.

## Results

Due to the differences in the surgical procedure, the patients that underwent the corrective meniscectomy were analysed separately and indicated in the text with the code “SM”; patients that underwent the chondral shaving only are indicated with the code “SH”. Background data of the two sub-populations included in the study are reported in Table 1.

**Table 1:** Characteristics of the 2 study populations

	SH group	MS group
Age		
Mean	53 y.o.	55 y.o.
SD	12	11
Gender		
M	13 (62%)	8 (57%)
F	8 (38%)	6 (43%)
BMI		
Mean	27	27
SD	4	4
Smoke		
Smokers/Former	8 (38%)	5 (36%)
Non Smokers	13 (62%)	9 (64%)
Grade Chondropathy (ICRS classification)		
II	5 (24%)	2 (14%)
III	8 (38%)	7 (50%)
IV	8 (38%)	5 (36%)
Grade OA (Kellgren Lawrence)		
1	8 (38%)	1 (7%)
2	4 (19%)	2 (14%)
3	9 (43%)	11 (79%)
Site of Lesion		
FC	10 (48%)	11 (79%)
PF	3 (14%)	3 (21%)
TP	8 (38%)	–
Type of Lesion		
Diffused	16 (76%)	11 (79%)
Focal - mean size	5 (24%) - 10 mm	3 (21%) - 14 mm
Surgery	SH	SM

*SD* standard deviation, *FC* femoral condyle, *PF* patellofemoral, *TP* tibial plateau, *SH* chondral shaving, *SM* chondral shaving + meniscectomy

**Fig. 1:**
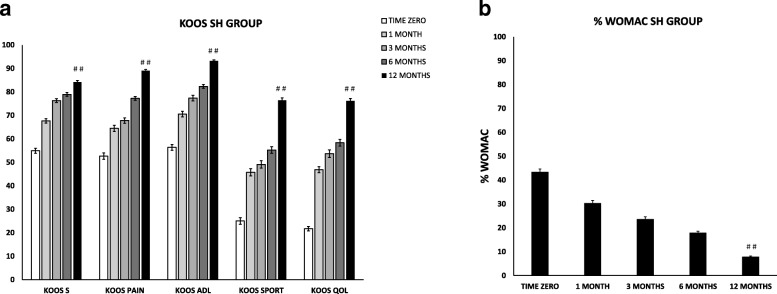
Trend of functional improvements of the SH group from baseline to 12 months’ follow-up. Results are expressed as mean and standard error. A *p* < 0.05 (T12 vs. T0) was considered statistically significant (# #). **a** KOOS score. KOOS S = symptoms; KOOS P = pain; KOOS ADL = activity daily living; KOOS Spt = sport; KOOS QoL = quality of life. **b** WOMAC Index

The analysis of the SH category data revealed a steady and statistically significant improvement of all the clinical scores from pre-operative evaluation to 1, 3, 6, and 12 months follow-up (Fig. 1a). At 12 months, the average improvement in KOOS compared to pre-operative condition was 29 in symptoms (*p* < 0.0001), 36 in pain (*p* < 0.0001), 37 in function in daily living (*p* < 0.0001), 51 in sport (p < 0.0001) and 54 in the quality of life (*p* < 0.0001). Statistically significant differences (*p* < 0.0001) between pre-treatment and follow-up values were found also for the total WOMAC scores. In details, pain, stiffness and functional limitation decreased from an average of 43 at baseline to 30 at 1 month, 24 at 3 months, 18 at 6 months, and 8 at 12 months (Fig. 1b). The physical examination at 6 months revealed that the majority of the patients entered in the category “normal” and, at 12 months, no patients were in the category “symptomatic” except for one patient in squatting (see Table 2 for comparison with the pre-operative condition).

**Table 2:** Knee objective evaluation – summary of the results

	ROM	PAT SUBLUX	Walking	Stairs	Squatting	Strength	Stiffness	Swelling
SH								
Pre-op								
N	43%	86%	76%	71%	5%	90%	95%	57%
S	57%	14%	24%	29%	95%	10%	5%	43%
6 months								
N	100%	100%	100%	100%	71%	100%	100%	100%
S	0%	0%	0%	0%	29%	0%	0%	0%
12 months								
N	100%	100%	100%	100%	95%	100%	100%	100%
S	0%	0%	0%	0%	5%	0%	0%	0%
MS								
Pre-op								
N	36%	100%	86%	43%	0%	100%	100%	36%
S	64%	0%	14%	57%	100%	0%	0%	64%
6 months								
N	100%	100%	100%	92%	77%	100%	100%	100%
S	0%	0%	0%	8%	23%	0%	0%	0%
12 months								
N	100%	100%	93%	100%	86%	100%	100%	100%
S	0%	0%	7%	0%	14%	0%	0%	0%

*N* normal, *S* symptomatic, *ROM* range of motion, *PAT SUBLUX* patellar subluxation

**Fig. 2:**
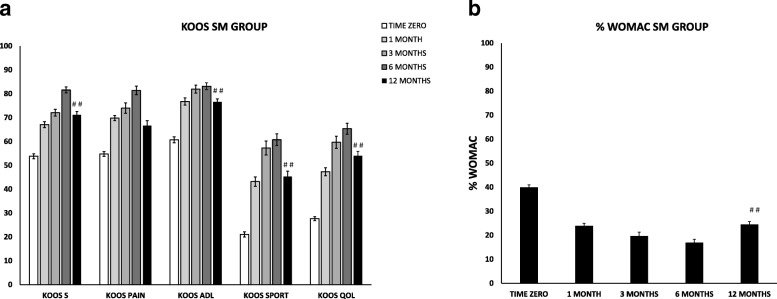
Trend of functional improvements of the SM group from baseline to 12 months’ follow-up. Results are expressed as mean and standard error. A *p* < 0.05 (T12 vs. T0) was considered statistically significant (# #). **a** KOOS score. KOOS S = symptoms; KOOS P = pain; KOOS ADL = activity daily living; KOOS Spt = sport; KOOS QoL = quality of life. **b** WOMAC Index

Results of the SM population appeared slightly different, with a steady and statistically significant improvement of all the clinical scores until the 6 months follow-up followed by a slight, but not statistically significant, decrease at 12 months (Fig. 2a). At 12 months, the average improvement in KOOS compared to pre-operative condition was 17 in symptoms (*p* = 0.014), 12 in pain (*p* = 0.183), 16 in function in daily living (*p* = 0.027), 24 in sport (*p* = 0.014) and 26 in the quality of life (*p* = 0.002). The same trend was found between pre-treatment and follow-up values for the total WOMAC scores (*p* < 0.0001). In details, pain, stiffness and functional limitation decreased from an average of 40 at baseline to 24 at 1 month, 20 at 3 months, 17 at 6 months, and 24 at 12 months (Fig. 2b). The physical examination at 6 months revealed that the majority of the patients entered in the category “normal” and, at 12 months, no patients were in the category “symptomatic” except for one patient in walking and two patients in squatting (see Table 2 for comparison with pre-operative condition). The comparison of the two populations revealed that SM patients improved less, but still a lot, compared to SH patients (Δ _t12-t0_ KOOS symptoms 17 vs. 29 [p≅0.05], pain 12 vs. 36 [*p* < 0.05], function in daily living 16 vs. 37 [p < 0.05], sport 24 vs. 51 [*p* < 0.05], and quality of life 26 vs. 54 [*p* < 0.01]). Interestingly, despite this finding, the Student’s t-test revealed that, besides the meniscectomy, other parameters such as age, sex, type and grade of chondropathy affect specific outcomes at different time points. Due to the small number of patients, this analysis has been performed on the whole population (35 patients). At 1 month, women and patients under 55 years old showed better improvements in the KOOS sport compared to men and elderly patients respectively (31% women vs. 14% men [*p* < 0.05] and 30% patients < 55 y.o. vs. 9% patients > 55 y.o. [*p* < 0.01]). At 3 months, patients with a chondropathy of grade IV showed higher improvements in the KOOS quality of life compared to patients with a grade II-III (51% grade IV vs. 31% grade II-III, p≅0.05). In addition, patients with a femoral condyle chondropathy showed better improvements in the KOOS pain compared to patients affected by a patellofemoral chondropathy (31% femoral condyle vs. 11% patellofemoral, p≅0.05). At 6 months, patients under 55 years old showed better improvements in the KOOS sport compared to elderly patients (41% vs. 23% respectively, p < 0.05). At 12 months, only the surgical procedure (meniscectomy), as already shown, affects the outcomes. Considering the improvement between the pre-operative scores and the last follow up at 12 months, females showed better improvements in KOOS functional score with respect to males (tendency, *p* = 0.077).

Interestingly, the degree of OA does not significantly affect the outcomes.

On average, 92% of the SH patients and 74% of the SM patients clinically improved (Table 3) and 100% of them were satisfied with the treatment.

**Table 3:** Summary of the results

	Δ KOOS_s	Δ KOOS_p	Δ KOOS_adl	Δ KOOS_spt	Δ KOOS_QoL	Δ WOMAC
SH						
Mean	29	36	37	51	54	−36
SE	1.11	1.45	1.31	1.73	1.32	1.31
% improved patients	90%	86%	95%	90%	95%	95%
SM						
Mean	17	12	16	24	26	−15
SE	2.2	2.4	1.7	2.7	1.7	1.8
% improved patients	64%	57%	86%	71%	86%	79%

Data are expressed as mean Δ (t_12_-t_0_) and standard error (SE)

*SH* chondral shaving, *SM* chondral shaving + meniscectomy, *s* symptoms, *p* pain, *adl* function in daily living, *spt* sport, *QoL* quality of life

No adverse events or complications were observed throughout the follow-up period, apart from one case of a temporary and small subcutaneous hematoma that did not require any additional treatment. For the sake of comprehensiveness, we report also the exceptional outcomes of the three patients (ICRS grade III-IV) that underwent the micro-perforations associated with micro-fragmented adipose tissue injection (data not shown). At 12 months, the average improvement for KOOS was 46 in symptoms (*p* < 0.0001), 48 in pain (*p* < 0.0001), 41 in function in daily living (*p* < 0.0001), 28 in sport (*p* < 0.0001) and 25 in the quality of life (*p* < 0.0001). Statistically significant differences (*p* < 0.0001) between pre-treatment and follow-up values were found also for the total WOMAC scores where the pain, stiffness and functional limitation decreased from 56 at baseline to 12 at 12 months follow-up.

## Discussion

This study retrospectively analysed the safety and potential benefits of using autologous and micro-fragmented adipose tissue as adjuvant in the surgical treatment of degenerative knee chondropathy. The results demonstrated that, when associated with a shaving procedure, it improves symptoms and function at least until 1-year follow-up, with a trend of steady increase during time. Indeed, a constant and statistically significant improvement of all the clinical scores was observed from pre-op evaluation to the 1, 3, 6, and 12 months follow-up with KOOS sport and quality of life being the most improved scores.

Articular cartilage lesions and degenerations, generally associated with disability and symptoms such as joint pain and reduced function, are hard to treat and remain challenging. Current pharmacologic interventions only temporarily reduce pain and symptoms, but no proven disease-modifying therapy is available [1]. Non-surgical treatments, such as HA or PRP injections target the symptoms but cannot inhibit the degeneration process. Joint-preserving surgical treatments, such as arthroscopic shaving, debridement, laser chondroplasty, and microfractures, provide temporary relief of symptoms, but for the diffused degenerative pathology or chondropathies at the initial stage are contraindicated by many authors. In these cases, regenerative medicine, and, in particular, the use of progenitor cells is preferable. Indeed, besides their multipotency, these cells secrete a variety of bioactive molecules that act in a paracrine manner to prime and sustain angiogenic, anti-fibrotic, anti-apoptotic, anti-microbial and immunomodulatory responses in the target tissue [9, 11, 36].

A very important finding of this study, in addition to the constant improvement of all the clinical scores from baseline evaluation to the 1, 3, 6, and 12 months follow-up, is that no patient worsened compared to the pre-operative condition or underwent additional treatments. Noteworthy, patients who declared to be very active in sport still improved, demonstrating that also high functional demands benefit from the treatment (data not shown). The meniscectomy did not substantially affected the outcomes that were, anyway, very positive, with an average improvements of all the KOOS scores of 20 points and a physical examination positive for all the patients except for 2 in squatting. This is a very important finding, considering reported data showing that meniscectomy in the long term has been shown both experimentally and clinically to exacerbate the osteoarthritic condition [1]. The observed decrease in the scores from 6 to 12 months, although not statistically significant, can be explained by a loss of the functional unit “meniscus” in terms of knee stability leading to a progressive chondral damage. Nevertheless, the MRI at 12 months revealed no signs of bone edema in the treated compartments as index of severe chondral damage. The main limitation of the study is the lack of a control group that does not allow for any definitive conclusion about micro-fragmented adipose tissue effect. Nevertheless, we believe that this approach is very promising since, in our experience, standard chondral shaving did not allow for satisfactory results so far.

## Conclusion

The results of this study show the safety and potential benefits of using autologous, micro-fragmented and minimally manipulated adipose tissue injection associated with the arthroscopic chondral shaving for the treatment of diffused knee chondropathy. The procedure is simple, sustainable, quick, minimally invasive, one-step, and safe. After one year, the results are very satisfactory and promising. A longer follow-up and a randomized controlled study on a big number of patients is needed to draw definitive conclusions and enlarge the indications.

## Abbreviations

ASCs: Adipose-derived stem cells
FC: Femoral condyle
HA: Hyaluronic acid
KOOS: Knee Injury and Osteoarthritis Outcome Score
MSCs: Mesenchymal stem cells
NSAIDs: Nonsteroidal anti-inflammatory drugs
OA: Osteoarthritis
PF: Patellofemoral
PRP: Platelet-rich plasma
SVF: Stromal vascular fraction
TP: Tibial plateau
VAS: Visual analogue pain scale
WOMAC: Western Ontario and McMaster Universities Osteoarthritis Index
